# Supplementary material for: Midlife and old-age cardiovascular risk factors, educational attainment, and cognition at 90-years – population-based study with 48-years of follow-up
Source: PLoS One. 2025 Oct 1;20(10):e0331385. doi: 10.1371/journal.pone.0331385 (PMC12488009; doi:10.1371/journal.pone.0331385)
Supplement: S1 Table — (DOCX) [file pone.0331385.s002.docx]

**S1 Table. Alcohol use in a month. Those who participated in telephone interviews and questionnaires at 90 years old. Highest reported frequency of use for any category of alcoholic beverage (beer, wine, or liquor).**

|  | **N** | | | |
| --- | --- | --- | --- | --- |
| **How often do you use alcoholic beverages?** | **1975** | **1981** | **1990** | **90 yrs.** |
| Never | 22 | 21 | 10 | 40 |
| Less than two days a month on average | 38 | 47 | 25 | 32 |
| 3-8 days / month | 24 | 13 | 13 | 17 |
| 9-16 days / month | 6 | 9 | 3 | 2 |
| over 16 days / month | 2 | 1 | 1 | 5 |

yrs. = years.
