# Supplementary material for: Midlife and old-age cardiovascular risk factors, educational attainment, and cognition at 90-years – population-based study with 48-years of follow-up
Source: PLoS One. 2025 Oct 1;20(10):e0331385. doi: 10.1371/journal.pone.0331385 (PMC12488009; doi:10.1371/journal.pone.0331385)
Supplement: S2 Table — (DOCX) [file pone.0331385.s003.docx]

**S2 Table. Linear regression analysis results for lifestyle factors at 90 years old predicting semantic fluency, immediate recall, delayed recall, and composite cognitive score at 90 years old.**

|  |  |  | **Semantic fluency** |  | **Immediate recall** |  | **Delayed recall** |  | **Composite score** |  |
| --- | --- | --- | --- | --- | --- | --- | --- | --- | --- | --- |
|  | **Risk factor** | **N** | **b (95%CI)** | ***p*** | **b (95%CI)** | ***p*** | **b (95%CI)** | ***p*** | **b (95%CI)** | ***p*** |
| **Model 1** | BP | 90 (89) | -3.01 (-5.24; -0.79) | 0.009 | -0.98 (-2.88; 0.93) | 0.312 | -0.13 (-0.59; 0.32) | 0.569 | -0.35 (-0.67; -0.02) | 0.040 |
|  | Chol | 66 (65) | 0.10 (-2.20; 2.41) | 0.929 | -2.34 (-4.69; 0.01) | 0.051 | -0.32 (-0.77; 0.13) | 0.159 | -0.24 (-0.62; 0.15) | 0.223 |
|  | BMI | 95 (94) | 0.07 (-0.19; 0.33) | 0.585 | 0 (-0.27; 0.27) | 0.999 | 0.03 (-0.03; 0.08) | 0.313 | 0.00 (-0.03; 0.04) | 0.805 |
|  | MET | 84 (83) | -0.09 (-0.68; 0.50) | 0.765 | -0.22 (-0.84; 0.39) | 0.477 | -0.08 (-0.22; 0.07) | 0.306 | -0.04 (-0.13; 0.06) | 0.446 |
|  | Edu lev 1 | 96 (95) | 0.83 (-1.26; 2.91) | 0.432 | 2.63 (0.58; 4.68) | 0.013 | 0.49 (-0.01; 0.99) | 0.056 | 0.30 (-0.01; 0.61) | 0.054 |
|  | Edu lev 2 | 96 (95) | 4.47 (1.50; 7.44) | 0.004 | 5.36 (3.75; 6.98) | <0.001 | 1.11 (0.75; 1.46) | <0.001 | 1.09 (0.83; 1.34) | <0.001 |
|  |  |  |  |  |  |  |  |  |  |  |
| **Model 2** | BP | 90 (89) | -2.89 (-4.86; -0.91) | 0.005 | -0.74 (-2.43; 0.95) | 0.385 | -0.15 (-0.58; 0.28) | 0.484 | -0.31 (-0.58; -0.05) | 0.022 |
|  | Chol | 66 (65) | 0.41 (-1.79; 2.61) | 0.711 | -1.94 (-4.09; 0.21) | 0.076 | -0.29 (-0.70; 0.13) | 0.175 | -0.16 (-0.48; 0.16) | 0.319 |
|  | BMI | 95 (94) | 0.10 (-0.14; 0.34) | 0.399 | 0.04 (-0.20; 0.28) | 0.760 | 0.04 (-0.02; 0.09) | 0.197 | 0.01 (-0.02; 0.04) | 0.453 |
|  | MET | 84 (83) | -0.34 (-0.94; 0.26) | 0.265 | -0.53 (-1.09; 0.04) | 0.068 | -0.15 (-0.28; -0.02) | 0.020 | -0.09 (-0.17; -0.02) | 0.019 |
|  |  |  |  |  |  |  |  |  |  |  |
| **Model 3** | BP | 77 (76) | -3.41 (-5.58; -1.24) | 0.003 | -0.87 (-2.66; 0.93) | 0.339 | -0.12 (-0.69; 0.44) | 0.666 | -0.32 (-0.63; -0.02) | 0.039 |
|  | Chol | 56 (55) | 0.10 (-2.37; 2.57) | 0.934 | -2.19 (-4.68; 0.30) | 0.084 | -0.36 (-0.85; 0.13) | 0.154 | -0.20 (-0.57; 0.17) | 0.282 |
|  | BMI | 82 (81) | 0.14 (-0.10; 0.38) | 0.240 | 0.05 (-0.20; 0.30) | 0.681 | 0.04 (-0.02; 0.09) | 0.215 | 0.02 (-0.02; 0.05) | 0.332 |
|  | MET | 71 (70) | -0.31 (-0.98; 0.37) | 0.369 | -0.51 (-1.14; 0.12) | 0.113 | -0.18 (-0.33; -0.02) | 0.024 | -0.09 (-0.18; 0.00) | 0.051 |
|  | Edu lev 1* | 83 (82) | 0.96 (-1.28; 3.20) | 0.397 | 2.81 (0.65; 4.96) | 0.011 | 0.42 (-0.70; 0.93) | 0.111 | 0.31 (-0.03; 0.64) | 0.071 |
|  | Edu lev 2* | 83 (82) | 5.22 (2.27; 8.17) | 0.001 | 5.61 (3.49; 7.74) | <0.001 | 1.04 (0.63; 1.45) | <0.001 | 1.13 (0.82; 1.44) | <0.001 |
|  |  |  |  |  |  |  |  |  |  |  |

BMI = body mass index, BP = blood pressure, Chol = cholesterol, CI = confidence intervals, EDU lev 1 = education category 1 (7–11 years), EDU lev 2 = education category 2 (above 12 years), MET = metabolic equivalent hours per day. Model 1: Sex, and age (centered) are used as covariates. Model 2: Sex, age (centered), and education are used as covariates. Model 3: Sex, age (centered), education, and APOE are used as covariates. Analyses adjusted for non-independence of twin data. *Covariates for education in model 3 were sex, age (centered), and APOE status.
