# Supplementary material for: Midlife and old-age cardiovascular risk factors, educational attainment, and cognition at 90-years – population-based study with 48-years of follow-up
Source: PLoS One. 2025 Oct 1;20(10):e0331385. doi: 10.1371/journal.pone.0331385 (PMC12488009; doi:10.1371/journal.pone.0331385)
Supplement: S3 Table — (DOCX) [file pone.0331385.s004.docx]

**S3 Table. Linear regression analysis results for lifestyle factors in 1981 predicting semantic fluency, immediate recall, delayed recall, and composite cognitive score at 90 years old.**

|  |  |  | **Semantic fluency** |  | **Immediate recall** |  | **Delayed recall** |  | **Composite score** |  |
| --- | --- | --- | --- | --- | --- | --- | --- | --- | --- | --- |
|  | **Risk factor** | **N** | **b (95%CI)** | ***p*** | **b (95%CI)** | ***p*** | **b (95%CI)** | ***p*** | **b (95%CI)** | ***p*** |
| **Model 1** | BP | 85 (84) | 3.54 (0.58; 6.51) | 0.020 | 3.27 (0.06; 6.49) | 0.046 | 0.74 (0.17; 1.31) | 0.012 | 0.62 (0.14; 1.10) | 0.012 |
|  | Chol | 36 (35) | 1.59 (-1.81; 4.99) | 0.346 | 2.52 (-0.96; 6.01) | 0.150 | 0.15 (-0.55; 85) | 0.674 | 0.40 (-0.13; 0.93) | 0.135 |
|  | BMI | 91 (90) | 0.21 (-0.10; 0.51) | 0.181 | -0.09 (-0.50; 0.32) | 0.661 | 0 (-0.09; 0.08) | 0.977 | -0.01 (-0.06; 0.04) | 0.714 |
|  | MET | 86 (85) | 0.43 (-0.28; 1.14) | 0.230 | 0.17 (-0.52; 0.86) | 0.616 | 0.01 (-0.11; 0.14) | 0.842 | 0.06 (-0.05; 0.16) | 0.305 |
|  | Edu lev 1 | 91 (90) | 0.70 (-1.39; 2.78) | 0.509 | 1.52 (-0.86; 3.89) | 0.208 | 0.24 (-0.35; 0.83) | 0.419 | 0.14 (-0.20; 0.48) | 0.411 |
|  | Edu lev 2 | 91 (90) | 4.19 (1.29; 7.08) | 0.005 | 4.77 (2.88; 6.66) | <0.001 | 0.99 (0.60; 1.39) | <0.001 | 0.99 (0.69; 1.28) | <0.001 |
|  |  |  |  |  |  |  |  |  |  |  |
| **Model 2** | BP | 85 (84) | 2.94 (0.18; 5.70) | 0.037 | 2.61 (-0.68; 5.89) | 0.118 | 0.68 (0.05; 1.30) | 0.036 | 0.46 (0.05; 0.87) | 0.027 |
|  | Chol | 36 (35) | 2.09 (-1.42; 5.60) | 0.233 | 3.32 (-0.98; 7.61) | 0.126 | 0.39 (-0.26; 1.04) | 0.236 | 0.51 (-0.12; 1.14) | 0.111 |
|  | BMI | 91 (90) | 0.30 (0.01; 0.59) | 0.044 | 0.01 (-0.42; 0.44) | 0.957 | 0.02 (-0.07; 0.11) | 0.649 | 0.01 (-0.05; 0.06) | 0.747 |
|  | MET | 86 (85) | 0.28 (-0.38; 0.95) | 0.400 | -0.06 (-0.75; 0.62) | 0.851 | -0.06 (-0.19; 0.07) | 0.356 | 0.02 (-0.08; 0.11) | 0.701 |
|  |  |  |  |  |  |  |  |  |  |  |
| **Model 3** | BP | 76 (75) | 2.95 (-0.02; 5.92) | 0.052 | 2.87 (-0.88; 6.62) | 0.131 | 0.67 (-0.10; 1.44) | 0.086 | 0.44 (-0.04; 0.93) | 0.074 |
|  | Chol | 34 (33) | 2.41 (-1.46; 6.27) | 0.213 | 2.96 (-1.15; 7.07) | 0.152 | 0.54 (-0.10; 1.17) | 0.097 | 0.50 (-0.10; 1.10) | 0.099 |
|  | BMI | 81 (80) | 0.34 (0.05; 0.62) | 0.020 | 0.08 (-0.40; 0.55) | 0.743 | 0.04 (-0.06; 0.13) | 0.425 | 0.02 (-0.04; 0.08) | 0.511 |
|  | MET | 77 (76) | 0.30 (-0.39; 0.99) | 0.392 | -0.11 (-0.85; 0.64) | 0.777 | -0.07 (-0.23; 0.09) | 0.380 | 0.02 (-0.08; 0.12) | 0.682 |
|  | Edu lev 1* | 81 (80) | 0.71 (-1.59; 3.02) | 0.540 | 1.51 (-1.04; 4.06) | 0.242 | 0.17 (-0.44; 0.77) | 0.591 | 0.12 (-0.26; 0.49) | 0.539 |
|  | Edu lev 2* | 81 (80) | 5.20 (2.57; 7.83) | <0.001 | 4.52 (2.23; 6.80) | <0.001 | 0.91 (0.47; 1.36) | <0.001 | 0.99 (0.66; 1.33) | <0.001 |
|  |  |  |  |  |  |  |  |  |  |  |

BMI = body mass index, BP = blood pressure, Chol = cholesterol, CI = confidence intervals, EDU lev 1 = education category 1 (7–11 years), EDU lev 2 = education category 2 (above 12 years), MET = metabolic equivalent hours per day. Model 1: Sex, age (centered) and follow-up time (centered) are used as covariates. Model 2: Sex, age (centered), follow-up time (centered), and education are used as covariates. Model 3: Sex, age (centered), follow-up time (centered), education, and APOE are used as covariates. BMI and MET variables are mean values based on 1975 and 1981 questionnaires. Analyses adjusted for non-independence of twin data. *Covariates for education in model 3 were sex, age (centered), follow-up time (centered), and APOE status.
