# Supplementary material for: Midlife and old-age cardiovascular risk factors, educational attainment, and cognition at 90-years – population-based study with 48-years of follow-up
Source: PLoS One. 2025 Oct 1;20(10):e0331385. doi: 10.1371/journal.pone.0331385 (PMC12488009; doi:10.1371/journal.pone.0331385)
Supplement: S4 Table — (DOCX) [file pone.0331385.s005.docx]

**S4 Table. Linear regression analysis results for lifestyle factors in 1990 predicting semantic fluency, immediate recall, delayed recall, and composite cognitive score at 90 years old.**

|  |  |  | **Semantic fluency** |  | **Immediate recall** |  | **Delayed recall** |  | **Composite score** |  |  |
| --- | --- | --- | --- | --- | --- | --- | --- | --- | --- | --- | --- |
| **Model 1** | **Risk factor** | **N** | **b (95%CI)** | ***p*** | **b (95%CI)** | ***p*** | **b (95%CI)** | ***p*** | **b (95%CI)** | ***p*** |  |
|  | BMI | 51 | 0.01 (-0.29; 0.31) | 0.937 | -0.22 (-0.55; 0.10) | 0.174 | -0.04 (-0.11; 0.03) | 0.223 | -0.03 (-0.08; 0.02) | 0.228 |  |
|  | BP | 49 | 1.63 (-1.77; 5.02) | 0.339 | 1.05 (-1.48; 3.58) | 0.405 | 0.22 (-0.25; 0.68) | 0.360 | 0.26 (-0.25; 0.76) | 0.308 |  |
|  | Chol | 42 | 2.19 (-1.28; 5.67) | 0.209 | 0.98 (-1.56; 3.53) | 0.438 | -0.11 (-0.64; 0.43) | 0.700 | 0.19 (-0.29; 0.67) | 0.429 | |
|  | MET | 52 | 0.22 (0.05; 0.38) | 0.010 | -0.03 (-0.33; 0.28) | 0.860 | 0.01 (-0.03; 0.05) | 0.700 | 0.02 (-0.02; 0.05) | 0.339 |  |
|  | Edu lev 1 | 53 | -0.21 (-3.27; 2.86) | 0.892 | 1.91 (-0.80; 4.62) | 0.163 | 0.07 (-0.46; 0.61) | 0.790 | 0.15 (-0.32; 0.63) | 0.516 |  |
|  | Edu lev 2 | 53 | 2.71 (-1.09; 6.51) | 0.157 | 3.97 (1.56; 6.38) | 0.002 | 0.58 (0.19; 0.97) | 0.004 | 0.72 (0.34; 1.10) | <0.001 |  |
|  |  |  |  |  |  |  |  |  |  |  |  |
| **Model 2** | BMI | 51 | 0.06 (-0.29; 0.41) | 0.737 | -0.11 (-0.42; 0.20) | 0.489 | -0.04 (-0.11; 0.04) | 0.370 | -0.02 (-0.07; 0.04) | 0.579 |  |
|  | BP | 49 | 1.46 (-1.53; 4.44) | 0.330 | 0.970 (-1.26; 3.25) | 0.377 | 0.17 (-0.30; 0.65) | 0.471 | 0.23 (-0.20; 0.66) | 0.279 |  |
|  | Chol | 42 | 1.41 (-3.14; 5.96) | 0.534 | 1.14 (-1.78; 4.05) | 0.434 | -0.28 (-0.87; 0.32) | 0.361 | 0.10 (-0.49; 0.70) | 0.722 |  |
|  | MET | 52 | 0.22 (0.04; 0.40) | 0.017 | -0.07 (-0.33; 0.20) | 0.604 | 0.003 (-0.03; 0.04) | 0.850 | 0.01 (-0.01; 0.04) | 0.325 |  |
|  |  |  |  |  |  |  |  |  |  |  |  |
| **Model 3** | BMI | 45 | 0.09 (-0.29; 0.47) | 0.638 | -0.13 (-0.47; 0.21) | 0.435 | -0.02 (-0.10; 0.06) | 0.632 | -0.01 (-0.07; 0.05) | 0.748 |  |
|  | BP | 44 | 1.464 (-2.067; 4.99) | 0.406 | 1.99 (-0.22; 4.20) | 0.077 | 0.36 (-0.11; 0.83) | 0.134 | 0.36 (-0.11; 0.84) | 0.131 |  |
|  | Chol | 37 | 0.64 (-3.94; 5.23) | 0.777 | 1.32 (-2.14; 4.77) | 0.443 | -0.31 (-0.94; 0.31) | 0.325 | 0.06 (-0.58; 0.71) | 0.840 |  |
|  | MET | 46 | 0.27 (0.08; 0.46) | 0.006 | -0.06 (-0.32; 0.19) | 0.620 | -0.004 (-0.04; 0.03) | 0.821 | 0.01 (-0.01; 0.04) | 0.268 |  |
|  | Edu lev 1* | 47 | -0.64 (-3.94; 2.66) | 0.696 | 2.23 (-0.98; 5.45) | 0.168 | 0.01 (-0.57; 0.59) | 0.973 | 0.12 (-0.42; 0.68) | 0.641 |  |
|  | Edu lev 2* | 47 | 3.87 (0.69; 7.05) | 0.019 | 3.96 (0.90; 7.01) | 0.012 | 0.44 (0.001; 0.89) | 0.049 | 0.74 (0.29; 1.18) | 0.002 |  |
|  |  |  |  |  |  |  |  |  |  |  |  |

BMI = body mass index, BP = blood pressure, Chol = cholesterol, CI = confidence intervals, EDU lev 1 = education category 1 (7–11 years), EDU lev 2 = education category 2 (above 12 years), MET = metabolic equivalent hours per day. Model 1: Sex, age (centered) and follow-up time (centered) are used as covariates. Model 2: Sex, age (centered), follow-up time (centered), and education are used as covariates. Model 3: Sex, age (centered), follow-up time (centered), education, and APOE are used as covariates. Analyses adjusted for non-independence of twin data. *Covariates for education in model 3 were sex, age (centered), follow-up time (centered), and APOE status.
