# Supplementary material for: Midlife and old-age cardiovascular risk factors, educational attainment, and cognition at 90-years – population-based study with 48-years of follow-up
Source: PLoS One. 2025 Oct 1;20(10):e0331385. doi: 10.1371/journal.pone.0331385 (PMC12488009; doi:10.1371/journal.pone.0331385)
Supplement: S5 Table — (DOCX) [file pone.0331385.s006.docx]

**S5 Table. Linear regression analysis results for lifestyle factors from 1975 predicting semantic fluency, immediate recall, delayed recall, and composite cognitive score at 90 years old.**

|  |  |  | **Semantic fluency** |  | **Immediate recall** |  | **Delayed recall** |  | **Composite score** |  |
| --- | --- | --- | --- | --- | --- | --- | --- | --- | --- | --- |
|  | **Risk factor** | **N** | **b (95%CI)** | ***p*** | **b (95%CI)** | ***p*** | **b (95%CI)** | ***p*** | **b (95%CI)** | ***p*** |
| **Model 1** | BP | 91 (90) | 3.03 (0.93; 5.14) | 0.005 | 3.01 (-0.13; 6.14) | 0.060 | 0.73 (0.23; 1.24) | 0.005 | 0.78 (0.29; 1.27) | 0.002 |
|  | EDU lev 1 | 93 (92) | 0.96 (-1.19; 3.11) | 0.378 | 2.97 (0.73; 5.21) | 0.010 | 0.56 (0.02; 1.11) | 0.042 | 0.35 (0.02; 0.67) | 0.039 |
|  | EDU lev 2 | 93 (92) | 4.16 (1.17; 7.15) | 0.007 | 5.55 (3.63; 7.46) | <0.001 | 1.19 (0.77; 1.60) | <0.001 | 1.10 (0.80; 1.39) | <0.001 |
|  |  |  |  |  |  |  |  |  |  |  |
| **Model 2** | BP | 91 (90) | 3.09 (0.43; 5.77) | 0.024 | 3.27 (0.33; 6.21) | 0.030 | 0.87 (0.41; 1.32) | <0.001 | 0.81 (0.32; 1.29) | 0.001 |
|  |  |  |  |  |  |  |  |  |  |  |
| **Model 3** | BP | 81 (80) | 4.24 (1.34; 7.14) | 0.005 | 3.90 (0.55; 7.25) | 0.023 | 0.87 (0.30; 1.43) | 0.003 | 0.92 (0.36; 1.49) | 0.002 |
|  | EDU lev 1* | 83 (82) | 0.93 (-1.35; 3.21) | 0.419 | 2.92 (0.56; 5.28) | 0.016 | 0.51 (-0.04; 1.06) | 0.067 | 0.32 (-0.02; 0.67) | 0.068 |
|  | EDU lev 2* | 83 (82) | 5.21 (2.47; 7.95) | <0.001 | 5.25 (2.89; 7.62) | <0.001 | 1.09 (0.63; 1.55) | <0.001 | 1.10 (0.76; 1.44) | <0.001 |
|  |  |  |  |  |  |  |  |  |  |  |

CI = confidence intervals. BP = blood pressure. EDU lev 1 = education category 1 (7-11 years), EDU lev 2 = education category 2 (above 12 years). Model 1: Sex, age (centered) and follow-up time (centered) are used as covariates. Model 2: Sex, age (centered), follow-up time (centered), and education are used as covariates. Model 3: Sex, age (centered), follow-up time (centered), education, and APOE are used as covariates. Analyses adjusted for non-independence of twin data. *Covariates for education in model 3 were sex, age (centered), follow-up time (centered), and APOE status.
