# Supplementary material for: Midlife and old-age cardiovascular risk factors, educational attainment, and cognition at 90-years – population-based study with 48-years of follow-up
Source: PLoS One. 2025 Oct 1;20(10):e0331385. doi: 10.1371/journal.pone.0331385 (PMC12488009; doi:10.1371/journal.pone.0331385)
Supplement: S6 Table — (DOCX) [file pone.0331385.s007.docx]

**S6 Table. Linear regression analysis results for midlife dementia risk scores, CAIDE and educational-occupational score, predicting semantic fluency, immediate recall, delayed recall, and composite cognitive score at 90 years old.**

|  |  |  | **Semantic fluency** |  | **Immediate recall** |  | **Delayed recall** |  | **Composite score** |  |
| --- | --- | --- | --- | --- | --- | --- | --- | --- | --- | --- |
|  | **Risk score** | **N** | **b (95%CI)** | ***p*** | **b (95%CI)** | ***p*** | **b (95%CI)** | ***p*** | **b (95%CI)** | ***p*** |
| **Model 1** | CAIDE total | 54 (53) | -0.01 (-0.92; 0.89) | 0.975 | 0.42 (-0.18; 1.01) | 0.169 | -0.002 (-0.15; 0.14) | 0.977 | 0.02 (-0.10; 0.14) | 0.765 |
|  | CAIDE (w/o EDU) | 54 (53) | 1.05 (0.05; 2.05) | 0.040 | 1.32 (0.44; 2.19) | 0.004 | 0.16 (-0.07; 0.39) | 0.186 | 0.21 (0.04; 0.37) | 0.015 |
|  | EDU-OCU | 94 (93) | 0.47 (0.13; 0.82) | 0.008 | 0.54 (0.31; 0.78) | <0.001 | 0.13 (0.08; 0.18) | <0.001 | 0.12 (0.08; 0.15) | <0.001 |
|  | EDU-OCU  (+ CAIDE w/o EDU) | 54 (53) | 0.65 (0.18; 1.11) | 0.007 | 0.37 (0.10; 0.64) | 0.009 | 0.12 (0.05; 0.20) | 0.002 | 0.11 (0.06; 0.16) | <0.001 |
| **Model 2** | CAIDE total | 47 (46) | -0.11 (-1.02; 0.81) | 0.817 | 0.60 (-0.04; 1.25) | 0.066 | 0.03 (-0.12; 0.19) | 0.661 | 0.04 (-0.10; 0.17) | 0.573 |
|  | CAIDE (w/o EDU) | 47 (46) | 0.85 (-0.15; 1.86) | 0.094 | 1.40 (0.51; 2.28) | 0.003 | 0.18 (-0.06; 0.42) | 0.150 | 0.20 (0.03; 0.37) | 0.021 |
|  | EDU-OCU | 83 (82) | 0.55 (0.19; 0.90) | 0.003 | 0.50 (0.20; 0.80) | 0.001 | 0.12 (0.06; 0.18) | <0.001 | 0.11 (0.07; 0.16) | <0.001 |
|  | EDU-OCU  (+ CAIDE w/o EDU) | 47 (46) | 0.81 (0.23; 1.38) | 0.007 | 0.34 (-0.03; 0.70) | 0.070 | 0.13 (0.03; 0.24) | 0.010 | 0.11 (0.04; 0.18) | 0.002 |

CAIDE = Cardiovascular Risk Factors, Aging and Dementia score, CI = confidence intervals, EDU = education, EDU-OCU = educational-occupational score, w/o = without. Model 1: Follow-up time (centered) used as a covariate. Model 2: Follow-up time (centered), and APOE are used as covariates. Analyses adjusted for non-independence of twin data.
