# Supplementary material for: Midlife and old-age cardiovascular risk factors, educational attainment, and cognition at 90-years – population-based study with 48-years of follow-up
Source: PLoS One. 2025 Oct 1;20(10):e0331385. doi: 10.1371/journal.pone.0331385 (PMC12488009; doi:10.1371/journal.pone.0331385)
Supplement: S7 Table — (DOCX) [file pone.0331385.s008.docx]

**S7A Table. Discordant twin pairs for semantic fluency and midlife BMI (cutoff 25).**

|  | **Control (lower BMI)** | |
| --- | --- | --- |
| **Case (higher BMI)** | Poorer memory | Better memory |
| Poorer memory | - | 2 |
| Better memory | 5 | - |

BMI = body mass index.

**S7B Table. Discordant twin pairs for semantic fluency and late midlife BMI (cutoff 25).**

|  | **Control (lower BMI)** | |
| --- | --- | --- |
| **Case (higher BMI)** | Poorer memory | Better memory |
| Poorer memory | - | 2 |
| Better memory | 4 | - |

BMI = body mass index.

**S7C Table. Discordant twin pairs for semantic fluency and old age BMI (cutoff 25).**

|  | **Control (lower BMI)** | |
| --- | --- | --- |
| **Case (higher BMI)** | Poorer memory | Better memory |
| Poorer memory | - | 2 |
| Better memory | 5 | - |

BMI = body mass index.

**S7D Table. Discordant twin pairs for semantic fluency and midlife physical activity.**

|  | **Control (higher MET)** | |
| --- | --- | --- |
| **Case (lower MET)** | Poorer memory | Better memory |
| Poorer memory | - | 4 |
| Better memory | 8 | - |

MET = metabolic equivalent hours per day.

**S7E Table. Discordant twin pairs for semantic fluency and late midlife physical activity (1990).**

|  | **Control (higher MET)** | |
| --- | --- | --- |
| **Case (lower MET)** | Poorer memory | Better memory |
| Poorer memory | - | 3 |
| Better memory | 7 | - |

MET = metabolic equivalent hours per day.

**S7F Table. Discordant twin pairs for semantic fluency and old age physical activity.**

|  | **Control (higher MET)** | |
| --- | --- | --- |
| **Case (lower MET)** | Poorer memory | Better memory |
| Poorer memory | - | 5 |
| Better memory | 4 | - |

MET = metabolic equivalent hours per day.

**S7G Table. Discordant twin pairs for semantic fluency and educational-occupational score.**

|  | **Control (higher EDU-OCU)** | |
| --- | --- | --- |
| **Case (lower EDU-OCU)** | Poorer memory | Better memory |
| Poorer memory | - | 2 |
| Better memory | 6 | - |

EDU-OCU = educational occupational score.
