# Supplementary material for: Midlife and old-age cardiovascular risk factors, educational attainment, and cognition at 90-years – population-based study with 48-years of follow-up
Source: PLoS One. 2025 Oct 1;20(10):e0331385. doi: 10.1371/journal.pone.0331385 (PMC12488009; doi:10.1371/journal.pone.0331385)
Supplement: S8 Table — (DOCX) [file pone.0331385.s009.docx]

**S8 Table. Descriptive statistics for those who participated in telephone interviews and questionnaires in 90 years old, and those who only participated in questionnaire at age 90.**

| **Characteristics** | **N** | | **All** | **Men** | **Women** |
| --- | --- | --- | --- | --- | --- |
| **All (90 yrs.)** |  | | **(96)** | **(41)** | **(55)** |
| **No telephone interview (90 yrs.)** |  | | **(91)** | **(21)** | **(70)** |
|  |  | | Mean (SD) | Mean (SD) | Mean (SD) |
| **Age** (years) |  | |  |  |  |
| 1975 | 92 | | 45.42 (2.27) | 45.84 (2.33) | 45.13 (2.21) |
| 1975 w/o telephone interview | 84 | | 44.74 (2.43) | 44.26 (1.69) | 44.88 (2.60) |
| 1981 | 90 | | 51.80 (2.43) | 52.08 (2.26) | 51.59 (2.23) |
| 1981 w/o telephone interview | 84 | | 51.05 (2.43) | 50.70 (1.89) | 51.15 (2.57) |
| 1990 | 53 | | 59.27 (1.11) | 59.31 (1.11) | 59.25 (1.12) |
| 1990 w/o telephone interview | 49 | | 58.74 (1.23) | 58.55 (1.45) | 58.80 (1.18) |
| 2020-2023 | 96 | | 91.22 (1.93) | 91.27 (1.95) | 91.20 (1.93) |
| 2020-2023 w/o telephone interview | 90 | | 90.98 (1.79) | 90.5 (0.95) | 91.11 (1.95) |
| **BMI** (kg/m^2^) |  | |  |  |  |
| 1975/1981 | 94 | | 24.67 (3.05) | 24.77 (2.46) | 24.60 (3.44) |
| 1975/1981 w/o telephone interview | 90 | | 24.22 (2.65) | 24.83 (2.26) | 24.04 (2.74) |
| 1990 | 51 | | 25.78 (3.72) | 25.43 (3.09) | 25.97 (4.06) |
| 1990 w/o telephone interview | 48 | | 25.28(2.96) | 25.57 (2.29) | 25.14 (3.21) |
| 2020-2023 | 95 | | 25.15 (4.10) | 24.56 (3.50) | 25.60 (4.48) |
| 2020-2023 w/o telephone interview | 83 | | 24.49 (4.13) | 25.11 (4.11) | 24.29 (4.14) |
| **Blood pressure** (high/normal) |  | |  |  |  |
| 1975 | 91 | | 6/85 | 2/36 | 4/49 |
| 1975 w/o telephone interview | 83 | | 11/72 | 4/14 | 7/58 |
| 1981 | 85 | | 13/72 | 4/34 | 9/38 |
| 1981 w/o telephone interview | 77 | | 21/56 | 6/10 | 15/46 |
| 1990 | 49 | | 11/38 | 3/14 | 8/24 |
| 1990 w/o telephone interview | 45 | | 21/24 | 4/6 | 17/18 |
| 2020-2023 | 90 | | 63/27 | 22/17 | 41/10 |
| 2020-2023 w/o telephone interview | 86 | | 54/32 | 10/11 | 44/21 |
| **Cholesterol** (high/normal) |  | |  |  |  |
| 1981 | 36 | | 7/29 | 5/18 | 2/11 |
| 1981 w/o telephone interview | 22 | | 7/15 | 3/4 | 4/11 |
| 1990 | 42 | | 23/19 | 6/10 | 17/9 |
| 1990 w/o telephone interview | 30 | | 14/16 | 2/4 | 12/12 |
| 2020-2023 | 65 | | 31/34 | 14/17 | 17/17 |
| 2020-2023 w/o telephone interview | 55 | | 17/38 | 6/11 | 11/27 |
| **Physical activity** (MET hours/day) |  | |  |  |  |
| 1975/1981 | 88 | | 2.40 (1.71) | 2.74 (1.85) | 2.14 (1.57) |
| 1975/1981 w/o telephone interview | 86 | | 1.96 (1.54) | 1.98 (1.68) | 1.95 (1.51) |
| 1990 | 52 | | 3.24 (4.56) | 4.88 (7.15) | 2.36 (1.87) |
| 1990 w/o telephone interview | 47 | | 2.41(1.98) | 2.81 (2.66) | 2.30 (1.79) |
| 2020-2023 | 84 | | 1.70 (1.65) | 1.79 (1.48) | 1.64 (1.79) |
| 2020-2023 w/o telephone interview | 54 | | 1.61 (1.80) | 2.55 (2.24) | 1.28 (1.52) |
| **CAIDE total** | 54 | | 7.61 (1.92) | 7.57 (1.73) | 7.65 (2.13) |
| **CAIDE total** w/o telephone interview | 44 | | 8.89 (2.22) | 9.7 (2.26) | 8.65 (2.19) |
| **EDU-OCU** | 94 | | 16.77 (3.15) | 16.68 (3.28) | 16.83 (3.08) |
| **EDU-OCU** w/o telephone interview | 88 | | 15.08 (2.26) | 14.86 (2.24) | 15.15 (2.28) |
| **Education 1975** (≤6yrs/ 7-11 yrs./ ≥12 yrs.) | 82 | | 33/40/19 | 11/19/8 | 22/21/11 |
| **Education 1975** w/o telephone interview | 92 | | 55/24/3 | 10/8/0 | 45/16/3 |
| **Education 1981** (≤6yrs/ 7-11 yrs./ ≥12 yrs.) | 94 | | 33/41/20 | 13/18/9 | 20/23/11 |
| **Education 1981** w/o telephone interview | 89 | | 58/27/4 | 12/8/0 | 46/19/4 |
| **Education 90 yrs.** (≤6yrs/ 7-11 yrs./ ≥12 yrs.) | | 96 | 38/38/20 | 17/15/9 | 21/23/11 |
| **Education 90 yrs.** w/o telephone interview | 91 | | 62/25/4 | 15/6/0 | 47/19/4 |
| ***APOE* status** ( ε4 carrier/non-carrier) | 83 | | 20/65 | 5/31 | 15/34 |
| ***APOE* status** w/o telephone interview | 63 | | 17/46 | 5/9 | 12/37 |

APOE = apolipoprotein E, BMI = body mass index, CAIDE = Cardiovascular Risk Factors, Aging and Dementia score, EDU-OCU = educational-occupational score, MET = metabolic equivalent hours per day, SD = standard deviation, w/o = without, yrs.= years.
