# Supplementary material for: Midlife and old-age cardiovascular risk factors, educational attainment, and cognition at 90-years – population-based study with 48-years of follow-up
Source: PLoS One. 2025 Oct 1;20(10):e0331385. doi: 10.1371/journal.pone.0331385 (PMC12488009; doi:10.1371/journal.pone.0331385)
Supplement: S9 Table — (DOCX) [file pone.0331385.s010.docx]

**S9 Table. Two-sample t-test results, examining midlife and old age cardiovascular risk factors and risk scores in those who participated in telephone interview and questionnaire at 90 years old, those who only participated in questionnaire at age 90, and those who were invited but did not participate.**

|  | **N** | | | **Telephone interview & questionnaire vs. neither** | | | | **Telephone interview & questionnaire vs. questionnaire only** | | | | **Mean (SD)** | | |
| --- | --- | --- | --- | --- | --- | --- | --- | --- | --- | --- | --- | --- | --- | --- |
| **Risk factor** | tele & qtn | qtn only | neither | t | *p* | 95% CI low | 95% CI upper | t | *p* | 95% CI low | 95% CI upper | tele & qtn | qtn only | neither |
| 75/81 BMI | 94 | 90 | 500 | 0.47 | 0.639 | -0.52 | 0.85 | -1.06 | 0.289 | -1.31 | 0.39 | 24.67 (3.05) | 24.22 (2.65) | 24.84 (2.74) |
| 1990 BMI | 51 | 48 | 198 | 0.91 | 0.366 | -0.95 | 2.56 | -0.75 | 0.456 | -1.82 | 0.83 | 25.78 (3.72) | 25.28 (2.96) | 26.59 (10.34) |
| 90 v. BMI | 95 | 83 | - | - | - | - | - | -1.06 | 0.291 | -1.89 | 0.57 | 25.15 (4.10) | 24.49 (4.13) | - |
| 75/81 PA | 88 | 86 | 469 | -0.28 | 0.777 | -0.48 | 0.36 | -1.75 | 0.082 | -0.94 | 0.06 | 2.40 (1.71) | 1.96 (1.54) | 2.34 (2.30) |
| 1990 PA | 52 | 47 | 193 | -0.69 | 0.493 | -1.88 | 0.91 | -1.15 | 0.254 | -2.26 | 0.61 | 3.24 (4.56) | 2.41 (1.98) | 2.75 (3.27) |
| 90 v. PA | 84 | 54 | - | - | - | - | - | -0.29 | 0.769 | -0.72 | 0.53 | 1.70 (1.65) | 1.61 (1.80) | - |
| EDU-OCU | 94 | 88 | 487 | -4.84 | <0.001 | -2.61 | -1.10 | -3.75 | <0.001 | -2.58 | -0.80 | 16.77 (3.15) | 15.08 (2.26) | 14.91 (2.63) |
| CAIDE | 54 | 44 | 210 | 2.50 | 0.013 | 0.17 | 1.40 | 2.82 | 0.006 | 0.38 | 2.17 | 7.61 (1.92) | 8.89 (2.22) | 8.40 (1.93) |
| CAIDE w/o EDU | 54 | 44 | 210 | 0.54 | 0.592 | -0.30 | 0.53 | 2.22 | 0.029 | 0.08 | 1.41 | 5.96 (1.36) | 6.70 (1.84) | 6.08 (1.57) |

BMI = body mass index, CAIDE = Cardiovascular Risk Factors, Aging and Dementia score, CI = confidence interval, EDU-OCU = educational-occupational score, edu = education, MET = metabolic equivalent hours per day, qtn = questionnaire, SD = standard deviation, tele = telephone interview, w/o = without. yrs. = years. Analyses adjusted for non-independence of twin data.
