# Supplementary material for: Midlife and old-age cardiovascular risk factors, educational attainment, and cognition at 90-years – population-based study with 48-years of follow-up
Source: PLoS One. 2025 Oct 1;20(10):e0331385. doi: 10.1371/journal.pone.0331385 (PMC12488009; doi:10.1371/journal.pone.0331385)
Supplement: S10 Table — (DOCX) [file pone.0331385.s011.docx]

**S10 Table. Desing based F-test results for midlife and old age risk factors in those who participated in telephone interview and questionnaire at 90 years old, those who only participated in questionnaire at age 90, and those who were invited but did not participate.**

|  | **N** | | | **Telephone interview & questionnaire vs. questionnaire only** | | | **Telephone interview & questionnaire vs. neither** | | |
| --- | --- | --- | --- | --- | --- | --- | --- | --- | --- |
| **Risk factor** | tele & qtn | qtn only | neither | Design-based F | df1, df2 | p | Design-based F | df1, df2 | p |
| BP 1975 | 91 | 83 | 458 | 2.18 | 1, 153 | 0.142 | 1.69 | 1, 463 | 0.195 |
| BP 1981 | 85 | 77 | 400 | 3.09 | 1, 142 | 0.081 | 3.69 | 1, 418 | 0.055 |
| BP 1990 | 49 | 45 | 181 | 6.70 | 1, 79 | 0.012 | 0.82 | 1, 190 | 0.367 |
| BP 90 yrs. | 90 | 86 | - | 0.99 | 1, 155 | 0.321 | - | - | - |
| Chol 1981 | 36 | 22 | 157 | 1.17 | 1, 54 | 0.284 | 0.06 | 1, 180 | 0.813 |
| Chol 1990 | 42 | 30 | 122 | 0.43 | 1, 63 | 0.514 | 0.64 | 1, 144 | 0.426 |
| Chol 90 yrs. | 65 | 55 | - | 3.16 | 1, 110 | 0.078 | - | - | - |
| EDU 1975 | 92 | 82 | 466 | 9.55 | 2.00, 307.73 | <0.001 | 19.19 | 2.00, 941.60 | <0.001 |
| EDU 1981 | 94 | 89 | 501 | 8.05 | 1.96, 314. 95 | <0.001 | 19.59 | 2.00, 1005. 83 | <0.001 |
| EDU 90 yrs. | 96 | 91 | - | 7.82 | 1.94, 318.05 | <0.001 | - | - | - |

BP = blood pressure, Chol = cholesterol, df = degrees of freedom, EDU = education, qtn = questionnaire, tele = telephone interview. yrs. = years. Analyses adjusted for non-independence of twin data.
