# Supplementary material for: Midlife and old-age cardiovascular risk factors, educational attainment, and cognition at 90-years – population-based study with 48-years of follow-up
Source: PLoS One. 2025 Oct 1;20(10):e0331385. doi: 10.1371/journal.pone.0331385 (PMC12488009; doi:10.1371/journal.pone.0331385)
Supplement: S11 Table — (DOCX) [file pone.0331385.s012.docx]

**S11 Table. Inverse probability weighted linear regression analysis results for lifestyle factors at 90 years old predicting semantic fluency, immediate recall, delayed recall, and compositive cognitive score at 90 years old.**

|  |  |  | **Semantic fluency** |  | **Immediate recall** |  | **Delayed recall** |  | **Composite score** |  |
| --- | --- | --- | --- | --- | --- | --- | --- | --- | --- | --- |
|  | **Risk factor** | **N** | **b (95%CI)** | ***p*** | **b (95%CI)** | ***p*** | **b (95%CI)** | ***p*** | **b (95%CI)** | ***p*** |
| **Model 1** | BP | 88 (87) | -2.33 (-4.72; 0.06) | 0.055 | -1.49 (-3.37; 0.39) | 0.199 | -0.20 (-0.77; 0.36) | 0.485 | -0.35 (-0.68; -0.01) | 0.041 |
|  | Chol | 64 (63) | 0.98 (-1; 22; 3.18) | 0.375 | -1.58 (-4.25; 1.09) | 0.242 | -0.35; -0.88; 0.19) | 0.205 | -0.10 (-0.48; 0.28) | 0.603 |
|  | BMI | 93 (92) | 0.12 (-0.11; 0.35) | 0.290 | -0.06 (-0.34; 0.23) | 0.693 | 0.03 (-0.04; 0.09) | 0.416 | 0.00 (-0.03; 0.04) | 0.858 |
|  | MET | 82 (81) | -0.08 (-0.66; 0.50) | 0.791 | -0.24 (-0.87; 0.39) | 0.448 | -0.21 (-0.38; -0.05) | 0.012 | -0.06 (-0.15; 0.02) | 0.136 |
|  | Edu lev 1 | 94 (93) | 1.24 (-0.89; 3.37) | 0.250 | 2.98 (0.95; 5.00) | 0.005 | 0.53 (-0.02; 1.08) | 0.061 | 0.36 (0.05; 0.68) | 0.025 |
|  | Edu lev 2 | 94 (93) | 4.25 (1.05; 7.45) | 0.010 | 5.98 (4.43; 7.54) | <0.001 | 1.13 (0.73; 1.54) | <0.001 | 1.12 (0.85; 1.40) | <0.001 |
|  |  |  |  |  |  |  |  |  |  |  |
| **Model 2** | BP | 88 (87) | -2.31 (-4.54; -0.07) | 0.043 | -1.42 (-3.06; 0.22) | 0.089 | -0.24 (-0.81; 0.33) | 0.405 | -0.34 (-0.63; -0.05) | 0.022 |
|  | Chol | 64 (63) | 1.08 (-1;04; 3.20) | 0.311 | -1.43 (-3.76; 0.90) | 0.224 | -0.35 (-0.87; 0.18) | 0.194 | -0.08 (-0.42; 0.26) | 0.634 |
|  | BMI | 93 (92) | 0.14 (-0.08; 0.37) | 0.216 | -0.02 (-0.27; 0.23) | 0.885 | 0.04 (-0.03; 0.10) | 0.251 | 0.01 (-0.02; 0.04) | 0.541 |
|  | MET | 82 (81) | -0.30 (-0.93; 0.33) | 0.345 | -0.54 (-1.19; 0.11) | 0.103 | -0.28 (-0.45; -0.12) | 0.001 | -0.11 (-0.20; -0.02) | 0.012 |
|  |  |  |  |  |  |  |  |  |  |  |
| **Model 3** | BP | 77 (76) | -2.29 (-4.60; 0.02) | 0.052 | -1.02 (-2.77; 0.74) | 0.253 | -0.25 (-0.87; 0.36) | 0.421 | -0.30 (-0.61; 0.01) | 0.059 |
|  | Chol | 56 (55) | 0.50 (-1.76; 2.76) | 0.658 | -1.75 (-4.17; 0.67) | 0.153 | -0.33 (-0.91; 0.25) | 0.264 | -0.15 (-0.50; 0.21) | 0.417 |
|  | BMI | 82 (81) | 0.20 (-0.03; 0.42) | 0.090 | -0.01 (-0.26; 0.24) | 0.925 | 0.03 (-0.03; 0.10) | 0.340 | 0.01 (-0.02; 0.04) | 0.403 |
|  | MET | 71 (70) | -0.24 (-0.91; 0.43) | 0.473 | -0.48 (-1.16; 0.20) | 0.162 | -0.30 (-0.48; -0.12) | 0.001 | -0.10 (-0.19; -0.01) | 0.033 |
|  | Edu lev 1* | 83 (82) | 1.37 (-1.08; 3.82) | 0.269 | 3.03 (0.88; 5.17) | 0.006 | 0.41 (-0.15; 0.98) | 0.149 | 0.35 (-0.00; 0.70) | 0.052 |
|  | Edu lev 2* | 83 (82) | 5.41 (2.49; 8.34) | <0.001 | 5.91 (3.84; 7.98) | <0.001 | 1.06 (0.60; 1.52) | <0.001 | 1.17 (0.85; 1.48) | <0.001 |
|  |  |  |  |  |  |  |  |  |  |  |

BMI = body mass index, BP = blood pressure, Chol = cholesterol, CI = confidence intervals, EDU lev 1 = education category 1 (7–11 years), EDU lev 2 = education category 2 (above 12 years), MET = metabolic equivalent hours per day. Model 1: Sex, and age (centered) are used as covariates. Model 2: Sex, age (centered), and education are used as covariates. Model 3: Sex, age (centered), education, and APOE are used as covariates. Analyses adjusted for non-independence of twin data. *Covariates for education in model 3 were sex, age (centered), follow-up time (centered), and APOE status.
