# Supplementary material for: Midlife and old-age cardiovascular risk factors, educational attainment, and cognition at 90-years – population-based study with 48-years of follow-up
Source: PLoS One. 2025 Oct 1;20(10):e0331385. doi: 10.1371/journal.pone.0331385 (PMC12488009; doi:10.1371/journal.pone.0331385)
Supplement: S12 Table — (DOCX) [file pone.0331385.s013.docx]

**S12 Table. Inverse probability weighted linear regression analysis results for lifestyle factors in 1981 predicting semantic fluency, immediate recall, delayed recall, and compositive cognitive score at 90 years old.**

|  |  |  | **Semantic fluency** |  | **Immediate recall** |  | **Delayed recall** |  | **Composite score** |  |
| --- | --- | --- | --- | --- | --- | --- | --- | --- | --- | --- |
|  | **Risk factor** | **N** | **b (95%CI)** | ***p*** | **b (95%CI)** | ***p*** | **b (95%CI)** | ***p*** | **b (95%CI)** | ***p*** |
| **Model 1** | BP | 85 (84) | 3.43 (0.34; 6.52) | 0.030 | 3.15 (-0.73; 7.03) | 0.110 | 0.85 (0.07; 1.63) | 0.032 | 0.56 (0.05; 1.08) | 0.032 |
|  | Chol | 36 (35) | 0.76 (-3.36; 4.88) | 0.710 | 4.17 (0.85; 7.50) | 0.015 | 0.50 (-0.48; 1.48) | 0.315 | 0.56 (0.02; 1.09) | 0.041 |
|  | BMI | 91 (90) | 0.29 (0.06; 0.52) | 0.015 | -0.07 (-0.43; 0.30) | 0.709 | 0.01 (-0.07; 0.10) | 0.780 | 0.00 (-0.04; 0.05) | 0.891 |
|  | MET | 86 (85) | 0.37 (-0.35; 1.09) | 0.307 | 0.13 (-0.57; 0.83) | 0.718 | 0.00 (-0.16; 0.16) | 0.975 | 0.04 (-0.06; 0.15) | 0.411 |
|  | Edu lev 1 | 91 (90) | 0.62 (-1.51; 2.76) | 0.564 | 1.19 (-1.43; 3.82) | 0.368 | 0.23 (-0.44; 0.90) | 0.499 | 0.11 (-0.26; 0.47) | 0.569 |
|  | Edu lev 2 | 91 (90) | 4.02 (0.91; 7.13) | 0.012 | 4.79 (2.95; 6.63) | <0.001 | 1.02 (0.59; 1.44) | <0.001 | 0.99 (0.68; 1.29) | <0.001 |
|  |  |  |  |  |  |  |  |  |  |  |
| **Model 2** | BP | 85 (84) | 3.11 (0.07; 6.15) | 0.045 | 2.63 (-1.32; 6.58) | 0.189 | 0.81 (-0.01; 1.62) | 0.053 | 0.47 (-0.04; 0.97) | 0.071 |
|  | Chol | 36 (35) | 1.03 (-3.07; 5.14) | 0.612 | 4.61 (1.18; 8.04) | 0.010 | 0.73 (-0.32; 1.78) | 0.172 | 0.61 (0.08; 1.15) | 0.026 |
|  | BMI | 91 (90) | 0.34 (0.11; 0.57) | 0.004 | -0.01 (-0.39; 0.37) | 0.959 | 0.02 (-0.06; 0.11) | 0.578 | 0.01 (-0.03; 0.06) | 0.574 |
|  | MET | 86 (85) | 0.23 (-0.47; 0.93) | 0.513 | -0.12 (-0.95; 0.71) | 0.768 | -0.08 (-0.26; 0.11) | 0.411 | 0.01 (-0.10; 0.12) | 0.894 |
|  |  |  |  |  |  |  |  |  |  |  |
| **Model 3** | BP | 76 (75) | 3.33 (-0.07; 6.73) | 0.055 | 2.37 (-1.92; 6.65) | 0.274 | 0.74 (-0.15; 1.63) | 0.102 | 0.43 (-0.14; 0.99) | 0.134 |
|  | Chol | 34 (33) | 1.44 (-3.38; 6.26) | 0.546 | 3.42 (-0.24; 7.08) | 0.066 | 0.47 (-0.38; 1.32) | 0.279 | 0.49 (-0.13; 1.10) | 0.115 |
|  | BMI | 81 (80) | 0.39 (0.14; 0.63) | 0.002 | 0.05 (-0.38; 0.49) | 0.802 | 0.04 (-0.04; 0.13) | 0.323 | 0.02 (-0.03; 0.08) | 0.340 |
|  | MET | 77 (76) | 0.15 (-0.60; 0.90) | 0.693 | -0.27 (-1.17; 0.64) | 0.560 | -0.11 (-0.32; 0.10) | 0.286 | -0.01 (-0.13; 0.10) | 0.808 |
|  | Edu lev 1* | 81 (80) | 0.73 (-1.76; 3.21) | 0.562 | 1.07 (-1.73; 3.88) | 0.447 | 0.10 (-0.59; 0.80) | 0.769 | 0.07 (-0.35; 0.49) | 0.743 |
|  | Edu lev 2* | 81 (80) | 5.20 (2.55; 7.85) | <0.001 | 4.52 (2.25; 6.79) | <0.001 | 0.94 (0.44; 1.44) | <0.001 | 1.00 (0.65; 1.36) | <0.001 |
|  |  |  |  |  |  |  |  |  |  |  |

BMI = body mass index, BP = blood pressure, Chol = cholesterol, CI = confidence intervals, EDU lev 1 = education category 1 (7–11 years), EDU lev 2 = education category 2 (above 12 years), MET = metabolic equivalent hours per day. Model 1: Sex, and age (centered) are used as covariates. Model 2: Sex, age (centered), and education are used as covariates. Model 3: Sex, age (centered), education, and APOE are used as covariates. Analyses adjusted for non-independence of twin data. *Covariates for education in model 3 were sex, age (centered), follow-up time (centered), and APOE status.
