# Supplementary material for: Midlife and old-age cardiovascular risk factors, educational attainment, and cognition at 90-years – population-based study with 48-years of follow-up
Source: PLoS One. 2025 Oct 1;20(10):e0331385. doi: 10.1371/journal.pone.0331385 (PMC12488009; doi:10.1371/journal.pone.0331385)
Supplement: S13 Table — (DOCX) [file pone.0331385.s014.docx]

**S13 Table. Inverse probability weighted linear regression analysis results for lifestyle factors in 1990 predicting semantic fluency, immediate recall, delayed recall, and compositive cognitive score at 90 years old.**

|  |  |  | **Semantic fluency** |  | **Immediate recall** |  | **Delayed recall** |  | **Composite score** |  |
| --- | --- | --- | --- | --- | --- | --- | --- | --- | --- | --- |
|  | **Risk factor** | **N** | **b (95%CI)** | ***p*** | **b (95%CI)** | ***p*** | **b (95%CI)** | ***p*** | **b (95%CI)** | ***p*** |
| **Model 1** | BP | 49 | 0.98 (-2.53; 4.48) | 0.577 | 1.05 (-1.25; 3.35) | 0.363 | 0.11 (-0.45; 0.67) | 0.700 | 0.17 (-0.31; 0.65) | 0.479 |
|  | Chol | 42 | 0.45 (-3.08; 3.99) | 0.797 | 0.27 (-2.74; 3.28) | 0.857 | -0.26 (-0.89; 0.36) | 0.406 | -0.03 (-0.58; 0.52) | 0.916 |
|  | BMI | 51 | 0.08 (-0.20; 0.36) | 0.581 | -0.20 (-0.52; 0.12) | 0.220 | -0.04 (-0.11; 0.02) | 0.211 | -0.02 (-0.07; 0.02) | 0.305 |
|  | MET | 52 | 0.24 (0.03; 0.45) | 0.028 | -0.04 (-0.36; 0.28) | 0.806 | 0.01 (-0.03; 0.05) | 0.748 | 0.02 (-0.02; 0.05) | 0.389 |
|  | Edu lev 1 | 53 | 0.28 (-2.84; 3.40) | 0.855 | 2.31 (-0.59; 5.22) | 0.116 | 0.03 (-0.55; 0.61) | 0.911 | 0.20 (-0.29; 0.70) | 0.414 |
|  | Edu lev 2 | 53 | 2.91 (-2.84; 3.40) | 0.146 | 4.69 (2.27; 7.12) | <0.001 | 0.58 (0.19; 0.97) | 0.004 | 0.80 (0.41; 1.18) | <0.001 |
|  |  |  |  |  |  |  |  |  |  |  |
| **Model 2** | BP | 49 | 0.91 (-2.46; 4.28) | 0.587 | 1.07 (-1.10; 3.23) | 0.325 | 0.08 (-0.49; 0.64) | 0.789 | 0.16 (-0.28; 0.60) | 0.462 |
|  | Chol | 42 | 0.26 (-4.07; 4.59) | 0.904 | 0.80 (-2.67; 4.26) | 0.644 | -0.36 (-1.03; 0.32) | 0.300 | -0.03 (-0.67; 0.62) | 0.936 |
|  | BMI | 51 | 0.13 (-0.19; 0.45) | 0.422 | -0.08 (-0.36; 0.20) | 0.565 | -0.04 (-0.11; 0.03) | 0.248 | -0.01 (-0.06; 0.04) | 0.641 |
|  | MET | 52 | 0.22 (0.01; 0.44) | 0.043 | -0.11 (-0.39; 0.17) | 0.450 | 0.00 (-0.03; 0.04) | 0.931 | 0.01 (-0.02; 0.04) | 0.561 |
|  |  |  |  |  |  |  |  |  |  |  |
| **Model 3** | BP | 44 | 1.12 (-2.63; 4.86) | 0.549 | 1.87 (-0.24; 3.99) | 0.081 | 0.24 (-0.36; 0.85) | 0.431 | 0.28 (-0.21; 0.76) | 0.257 |
|  | Chol | 37 | 0.02 (-4.91; 4.95) | 0.993 | 0.81 (-3.18; 4.79) | 0.683 | -0.25 (-0.91; 0.41) | 0.452 | -0.01 (-0.74; 0.72) | 0.980 |
|  | BMI | 45 | 0.13 (-0.22; 0.48) | 0.452 | -0.15 (-0.46; 0.16) | 0.347 | -0.02 (-0.10; 0.05) | 0.558 | -0.01 (-0.06; 0.04) | 0.701 |
|  | MET | 46 | 0.30 (0.09; 0.51) | 0.007 | -0.09 (-0.35; 0.18) | 0.510 | -0.01 (-0.04; 0.03) | 0.715 | 0.01 (-0.02; 0.04) | 0.368 |
|  | Edu lev 1* | 47 | -0.15 (-3.47; 3.17) | 0.928 | 2.74 (-0.61; 6.10) | 0.106 | -0.06 (-0.69; 0.57) | 0.856 | 0.18 (-0.39; 0.74) | 0.532 |
|  | Edu lev 2* | 47 | 4.42 (1.30; 7.54) | 0.007 | 4.81 (1.74; 7.89) | 0.003 | 0.42 (-0.04; 0.87) | 0.073 | 0.84 (0.37; 1.30) | 0.001 |
|  |  |  |  |  |  |  |  |  |  |  |

BMI = body mass index, BP = blood pressure, Chol = cholesterol, CI = confidence intervals, EDU lev 1 = education category 1 (7–11 years), EDU lev 2 = education category 2 (above 12 years), MET = metabolic equivalent hours per day. Model 1: Sex, and age (centered) are used as covariates. Model 2: Sex, age (centered), and education are used as covariates. Model 3: Sex, age (centered), education, and APOE are used as covariates. Analyses adjusted for non-independence of twin data. *Covariates for education in model 3 were sex, age (centered), follow-up time (centered), and APOE status.
