# Supplementary material for: Midlife and old-age cardiovascular risk factors, educational attainment, and cognition at 90-years – population-based study with 48-years of follow-up
Source: PLoS One. 2025 Oct 1;20(10):e0331385. doi: 10.1371/journal.pone.0331385 (PMC12488009; doi:10.1371/journal.pone.0331385)
Supplement: S14 Table — (DOCX) [file pone.0331385.s015.docx]

**S14 Table. Inverse probability weighted linear regression analysis results for lifestyle factors in 1975 predicting semantic fluency, immediate recall, delayed recall, and compositive cognitive score at 90 years old.**

|  |  |  | **Semantic fluency** |  | **Immediate recall** |  | **Delayed recall** |  | **Composite score** |  |
| --- | --- | --- | --- | --- | --- | --- | --- | --- | --- | --- |
|  | **Risk factor** | **N** | **b (95%CI)** | ***p*** | **b (95%CI)** | ***p*** | **b (95%CI)** | ***p*** | **b (95%CI)** | ***p*** |
| **Model 1** | BP | 91 (90) | 4.17 (1.50; 6.84) | 0.003 | 2.73 (-1.13; 6.59) | 0.163 | 0.87 (0.17; 1.57) | 0.015 | 0.81 (0.32; 1.31) | 0.002 |
|  | Edu lev 1 | 93 (92) | 1.03 (-1.10; 3.16) | 0.340 | 3.36 (1.10; 5.62) | 0.004 | 0.67 (0.09; 1.24) | 0.023 | 0.41 (0.06; 0.75) | 0.021 |
|  | Edu lev 2 | 93 (92) | 4.03 (0.91; 7.16) | 0.012 | 5.79 (3.89; 7.68) | <0.001 | 1.21 (0.76; 1.67) | <0.001 | 1.11 (0.81; 1.42) | <0.001 |
|  |  |  |  |  |  |  |  |  |  |  |
| **Model 2** | BP | 91 (90) | 4.19 (1.31; 7.07) | 0.005 | 2.84 (-0.55; 6.23) | 0.099 | 1.01 (0.34; 1.68) | 0.003 | 0.82 (0.36; 1.28) | 0.001 |
|  |  |  |  |  |  |  |  |  |  |  |
| **Model 3** | BP | 81 (80) | 4.90 (1.63; 8.18) | 0.004 | 2.87 (-0.94; 6.67) | 0.137 | 0.91 (0.19; 1.64) | 0.014 | 0.86 (0.33; 1.38) | 0.002 |
|  | Edu lev 1* | 83 (82) | 1.04 (-1.29; 3.36) | 0.376 | 3.30 (0.92; 5.68) | 0.007 | 0.59 (0.01; 1.17) | 0.046 | 0.38 (0.01; 0.74) | 0.042 |
|  | Edu lev 2* | 83 (82) | 5.22 (2.56; 7.89) | <0.001 | 5.52 (3.18; 7.87) | <0.001 | 1.13 (0.62; 1.64) | <0.001 | 1.13 (0.78; 1.48) | <0.001 |
|  |  |  |  |  |  |  |  |  |  |  |

BMI = body mass index, BP = blood pressure, Chol = cholesterol, CI = confidence intervals, EDU lev 1 = education category 1 (7–11 years), EDU lev 2 = education category 2 (above 12 years), MET = metabolic equivalent hours per day. Model 1: Sex, and age (centered) are used as covariates. Model 2: Sex, age (centered), and education are used as covariates. Model 3: Sex, age (centered), education, and APOE are used as covariates. Analyses adjusted for non-independence of twin data. *Covariates for education in model 3 were sex, age (centered), follow-up time (centered), and APOE status.
