# Supplementary material for: Midlife and old-age cardiovascular risk factors, educational attainment, and cognition at 90-years – population-based study with 48-years of follow-up
Source: PLoS One. 2025 Oct 1;20(10):e0331385. doi: 10.1371/journal.pone.0331385 (PMC12488009; doi:10.1371/journal.pone.0331385)
Supplement: S15 Table — (DOCX) [file pone.0331385.s016.docx]

**S15 Table. Inverse probability weighted linear regression analysis results for midlife dementia risk scores, CAIDE and educational-occupational score, predicting semantic fluency, immediate recall, delayed recall, and compositive cognitive score at 90 years old.**

|  |  |  | **Semantic fluency** |  | **Immediate recall** |  | **Delayed recall** |  | **Composite score** |  |
| --- | --- | --- | --- | --- | --- | --- | --- | --- | --- | --- |
|  | **Risk score** | **N** | **b (95%CI)** | ***p*** | **b (95%CI)** | ***p*** | **b (95%CI)** | ***p*** | **b (95%CI)** | ***p*** |
| **Model 1** | CAIDE total | 54 (53) | 0.29 (-0.52; 1.12) | 0.470 | 0.53 (-0.10; 1.16) | 0.095 | 0.05 (-0.12; 0.21) | 0.587 | 0.07 (-0.06; 0.19) | 0.277 |
|  | CAIDE (w/o EDU) | 54 (53) | 0.95 (0.01; 1.90) | 0.048 | 1.31 (0.44; 2.18) | 0.004 | 0.22 (-0.04; 0.48) | 0.095 | 0.21 (0.05; 0.36) | 0.013 |
|  | EDU-OCU | 94 (93) | 0.39 (-0.04; 0.82) | 0.077 | 0.46 (0.12; 0.80) | 0.008 | 0.13 (0.04; 0.21) | 0.005 | 0.09 (0.04; 0.14) | <0.001 |
|  | EDU-OCU  (+ CAIDE w/o EDU) | 54 (53) | 0.35 (-0.13; 0.82) | 0.146 | 0.35 (0.02; 0.69) | 0.039 | 0.13 (0.02; 0.24) | 0.026 | 0.07 (0.02; 0.13) | 0.014 |
| **Model 2** | CAIDE total | 47 (46) | 0.29 (-0.59; 1.16) | 0.509 | 0.71 (0.07; 1.35) | 0.030 | 0.08 (-0.08; 0.25) | 0.334 | 0.09 (-0.04; 0.22) | 0.170 |
|  | CAIDE (w/o EDU) | 47 (46) | 0.88 (-0.10; 1.87) | 0.078 | 1.36 (0.51; 2.20) | 0.002 | 0.23 (-0.02; 0.48) | 0.068 | 0.21 (0.05; 0.37) | 0.012 |
|  | EDU-OCU | 83 (82) | 0.44 (-0.05; 0.93) | 0.081 | 0.40 (0.01; 0.79) | 0.043 | 0.13 (0.03; 0.23) | 0.013 | 0.09 (0.03; 0.14) | 0.004 |
|  | EDU-OCU  (+ CAIDE w/o EDU) | 47 (46) | 0.43 (-0.23; 1.10) | 0.196 | 0.28 (-0.06; 0.63) | 0.101 | 0.14 (0.01; 0.27) | 0.031 | 0.07 (0.00 (0.14) | 0.049 |

CAIDE = Cardiovascular Risk Factors, Aging and Dementia score, CI = confidence intervals, EDU = education, EDU-OCU = educational-occupational score, w/o = without. Model 1: Follow-up time (centered) used as a covariate. Model 2: Follow-up time (centered), and APOE are used as covariates. Analyses adjusted for non-independence of twin data.
