# Supplementary material for: Midlife and old-age cardiovascular risk factors, educational attainment, and cognition at 90-years – population-based study with 48-years of follow-up
Source: PLoS One. 2025 Oct 1;20(10):e0331385. doi: 10.1371/journal.pone.0331385 (PMC12488009; doi:10.1371/journal.pone.0331385)
Supplement: Attachment — Submitted filename: Response_to_Reviewers.doc [file pone.0331385.s018.doc]

**Responses to reviewers**

We thank the reviewers and the editor for their insightful comments which have helped improve the manuscript. The changes in the manuscript are marked with Track Changes and by page number (referring to the document with track changes) in this response letter. The responses in this document are listed in the following order: comments by the editor, comments by reviewer #1, and comments by reviewer #2.

EDITORS COMMENTS:

1. If there are ethical or legal restrictions on sharing a de-identified data set, please explain them in detail (e.g., data contain potentially identifying or sensitive patient information, data are owned by a third-party organization, etc.) and who has imposed them (e.g., a Research Ethics Committee or Institutional Review Board, etc.). Please also provide contact information for a data access committee, ethics committee, or other institutional body to which data requests may be sent.

**Response:** Because of the consent given by the participants at the time of data collection, and the easier identification of twin data and sensitive nature of the questionnaire data, the data is not publicly available. Data are available through the Institute for Molecular Medicine Finland (FIMM) Data Access Committee (DAC), who have IRB/ethics approval and an institutionally approved study plan. This information and the contact information of the FIMM DAC (fimm-dac@helsinki.fi) is provided in the data availability statement in the manuscript.

2. If there are no restrictions, please upload the minimal anonymized data set necessary to replicate your study findings to a stable, public repository and provide us with the relevant URLs, DOIs, or accession numbers. Please see http://www.bmj.com/content/340/bmj.c181.long for guidelines on how to de-identify and prepare clinical data for publication. For a list of recommended repositories, please see https://journals.plos.org/plosone/s/recommended-repositories. You also have the option of uploading the data as Supporting Information files, but we would recommend depositing data directly to a data repository if possible.

Please update your Data Availability statement in the submission form accordingly.

**Response:** The restrictions for data availability are mentioned in the data availability statement, and contact information is provided.

3. Please include captions for your Supporting Information files at the end of your manuscript, and update any in-text citations to match accordingly. Please see our Supporting Information guidelines for more information: <http://journals.plos.org/plosone/s/supporting-information>.

**Response:** Captions for supporting information files have been added at the end of the manuscript file, and all in-text citations have been updated accordingly.

4. The research questions and analytic plan do not appear to have been pre-registered (e.g. Open Science Framework or similar). Given the number of statistical tests, this increases the likelihood of chance and unstable findings. Is there anywhere else the questions and plan were stated in advance (e.g. data sharing applications, ethics forms)? Include if possible.

**Response:** Unfortunately, the research plan was not pre-registered. A section was added to the manuscript (see page 18, discussion) mentioning the issue of multiple testing and how it could affect the observed associations.

5. There is no mention of childhood intelligence yet a substantial literature on the long-reaching consequences. Educational attainment is not the same thing, particularly for older cohorts and older women, whose years of education would be influenced by sociological factors not just childhood intelligence. At the very least, please acknowledge this literature and the limitation that you were not able to consider this in your analysis.

**Response**: The first paragraph of the discussion section (see page 16) was almost completely re-written to better describe how childhood intelligence and its long-reaching effects on cognitive function in old age relate to implication of our study results.

6. I was surprising not to see use of inverse probability weights to address non-random attrition and drop-out (and reduce bias). You could upweight participants similar to non-responders and dropouts, to reduce possible bias (or at least evaluate differences vs. unweighted analysis). This is fairly common an approach.

**Response:** We agree with this observation, and we have now conducted the inverse probability weighted analyses, and these are included in the revised manuscript. We used logistic regression to predict staying in the study based on sex and educational-occupational score, which differed significantly between participants and non-responders. The educational-occupational score also captures more information on socio-economic status than education alone. CAIDE score was not used due to the substantial reduction of the sample size for this variable. The regression analyses were then conducted again using stabilized inverse probability weights. The findings remained consistent with the original analyses and accordingly, these analyses did not change the conclusions of this study.

Tables with results have been added to the supplement (tables S17- S21), and following description of this analysis method was included in the main manuscript (page 8): “We also conducted an inverse probability weighting (IPW) analysis to adjust for potential bias due to non-random dropout. Logistic regression was used to estimate the probability of remaining in the study, with predictors including the educational-occupational score (which differed significantly between participants and non-participants), sex, and family relatedness (accounted for via clustering). Stabilized inverse probability weights were then applied in the regression models.”

Following text was added to the Result section (page 16): “Results from the IPW analyses are presented in the Supplement (Tables S17–S21). The findings remained consistent with the original analyses. The main difference observed in the IPW analyses was that the educational-occupational score was no longer significantly associated with semantic fluency, although it remained significantly associated with immediate recall, delayed recall, and the composite cognitive score.”

7. Please construct a composite of global/general function - all three tests, for comparison. I would have done global function as the main analysis, then specific tests as secondary or even supplementary/exploratory analysis.

**Response:** We agree with this suggestion, and we have constructed a composite cognitive score (mean of z-scores from each individual cognitive test) and used this in the revised manuscript. This outcome variable was included in all analyses, and the results added to the regression tables. A new figure was included for the composite cognitive score (Fig 5). The findings are mainly in line with the original findings. This additional analysis was incorporated in the manuscript text throughout.

8. The role of healthy survivor effects and very small sample size (low statistical power) are mentioned only briefly but are fundamental limitations. Much more emphasis and reflection on this is needed, particularly in the discussion section. Healthy survivor effects could create spurious and associations in the opposite direction to the causal effects.

**Response**: The limitations section in the Discussion has been almost completely re-written (pages 17-18), discussing the issues posed by the small sample size and the possible influence of the healthy survivor bias and reverse causation. In addition, more caution has been added to discussion of our results throughout the Discussion section.

P2 ABSTRACT. Is "Cardiovascular Risk Factors, Aging, and Dementia (CAIDE)" and "dementia risk factors" the same thing? Reword for clarity to avoid confusion.

**Response**: Added “individual” cardiovascular risk factors to more clearly make a distinction that we investigated the risk factors individually and together as the CAIDE score.

P2L36 Do you mean cognitive aging or aging?

**Response:** Corrected to “cognitive aging”

P2L40 "substantial proportion" and "high prevalence" reads awkwardly. Can you report prevalence for both with the same term?

**Response**: Removed “substantial proportion”

P3L54 "midlife and old age" is confusing - state age range more simply. Then there can be no doubt about the meaning of each term.

**Response**: Modified to include age range and refer to “very old age” rather than “old age.” The manuscript, page 3, now reads: “Our aim was to use a population-based sample of twins with up to 48 years of follow-up to investigate if CV risk factors measured in midlife (age range 42-51) and very old age (age range 90-98), including BMI, blood pressure (BP), cholesterol, and physical activity (PA) predict cognition at nonagenarian age.”

P3L56 It reads strangely that you are using "previously validated" dementia risk scores yet your question concerns their validity in a population they were not validated for. If the aim of the study is to validate them on an older age, be more clear about this. If the assumption is made that using risk scores validated at midlife will suffice on old age adults, that sounds quite weak, so needs rewriting for clarity and strength.

**Response**: This sentence was added for clarity (page 3): “While these scores were originally developed and validated to predict late-life dementia based on midlife risk factors, our aim here was to explore whether these midlife profiles remain predictive of cognitive outcomes at very old age.”

P3L58 Hypothesized - but not pre-registered anywhere?

**Response:** The study was not pre-registered.

P3L61 Missing statement "..have a reversed association with cognition ON THE BASIS THAT...."

**Response**: Added sentence (page 4): “…on the basis that in advanced stages of dementia, certain risk factors such as low BMI and hypertension may no longer indicate risk, but rather reflect the severity or progression of the disease itself [13].”

P3L65 I do not understand this: "irrespective of co-twin's vital status or participation". Do you mean any surviving twin was eligible? Please use the STROBE language (eligible, recruited, analyzed etc.) and add flow diagram.

**Response**: Added sentence (page 4): “All eligible surviving twins were recruited for the study, regardless of whether their co-twin was deceased or did not participate.” The data collection and waves of questionnaires are described in a flow diagram (Fig 1) that has been modified according to the comments in this review for more clarity.

P4 The NONAGINTA term suddenly appears from nowhere and is quite jarring. It needs a brief introduction and rewording e.g. to make it clear that this is a nested/sub study of a larger cohort.

**Response**: Sentence was modified to include better wording leading into the NONAGINTA term (page 4): “…and participated in a sub study focusing on twins turning 90-years-old, referred to as the NONAGINTA –study.”

P4L73 Why is the 1990 cohort so much smaller than the others?

**Response**: For the 1975 and 1981 questionnaires, all twins born prior to 1958 were invited for the mail-in questionnaire. In 1990, only twins born between 1930-1958 were invited, excluding those born before 1930, making the cohort smaller. A sentence was added/modified to clarify this in the methods section (page 4): “We also used earlier FTC data that were collected through postal questionnaires with a baseline in 1975 (all twins), and follow-ups in 1981 (all twins) and 1990 (only those born 1930 or later were invited).”

P4L81 Here and elsewhere, please use age range observed, and year(s) observed. Readers not involved in the study need to quickly understand how old and when data were collected.

**Response:** The age range was added to the text.

P4L89 Be careful creating binary variables, you lose variation and results are more likely to drift to null. See commentary on dichotomania (<https://www.fharrell.com/post/errmed/>)

**Response**: For blood pressure and cholesterol variables, which were based on self-reports and categorical variables, there were unfortunately too few participants in each group, and binary variables were the only option. Blood pressure and cholesterol measures were not available for the entire cohort at the earlier time-points.

P5L92 Language - "had used" not "have used". "none in 1981" not "while none in 1981"

**Response**: Corrected

P5L95 If you must used binary variables, simpler here to say normal/low and high rather than "One person...."

**Response**: Corrected

P5L103 Why frequency of alcohol use but not quantity or hazardous pattern?

**Response**: Majority of the participants reported frequency of alcohol use to be very low or none, which is common in the 90-year-old cohort. Based on this we determined frequency to be sufficient on its own to describe alcohol use in this cohort.

P5L107 Careful with the term "this study" which can be confusing when writing up specific questions in samples nested in wider cohorts. Some readers will get confused about what "study" this refers to, particularly if you have just mentioned three sets of questionnaires over many years. Consider "For our analysis" or similar.

**Response**: Changed to “for our analysis” everywhere where relevant.

P6L114 I don't understand this: "as opposed to original CAIDE with in-person measurements" and so might not readers.

**Response**: More detailed explanation was added to improve clarity (page 6): “CAIDE score was based on scores calculated in [8]. The score was determined from participants’ self-reports in 1975, 1981, and 1990 questionnaires, including BP, BMI (from height and weight), cholesterol levels, and exercise frequency. When data were missing at one time point, values from an alternate year or later questionnaire (e.g., 1990 for cholesterol) were used. The classification thresholds and scoring followed those of the original CAIDE model, with minor adjustments to accommodate available data. Original CAIDE was developed using in-person measurements [11].”

P6L113 I appreciate the need to reference previously published cut-points [8, 12, 8] but can you briefly state some additional information so that we don't have to look up and read these papers separately? It's a bit too brief currently.

**Response**: More detail was added to these paragraphs, especially information about the educational-occupational score (see pages 6-7). Revised text reads: “The educational-occupational score was based on the scores calculated in [12]. It consisted of the following self-reported variables from the 1975 (mean age 45) and 1981 (mean age 52) postal questionnaires: age, years of education, work status (not working, homemaker, or working/ studying), complexity of work (very monotonous, somewhat monotonous/variable, or very variable), physical loading of work (heavy manual labor, manual labor with lifting, light manual labor, and nonmanual work) and work environment (mainly outdoors or both indoors and outdoors, vs. indoors only) [8]. The questions for these variables and their categorization are explained in detail in [8].”

P6L145 I appreciate the drop-out analysis, but many readers (including me) are more familiar with inverse probability weighting to evaluate drop-out bias i.e. logistic regression of drop-out and end of follow-up based on socio-demographic and other predictors at baseline, save probability, take inverse, use inverse to upweight responders who look most similar to dropouts. Compare results side by side - materially different?

**Response:** Inverse probability weighting was conducted to evaluate drop-out bias. Detailed explanation of this can be found in the response for editor comment #6.

P6L153 Returning a questionnaire is widely considered to represent informed consent, particularly in an existing study where participants are used to completing them. Why would that differ today - can you briefly explain (I wasn't sure why newer legislation needs to be mentioned if it was ethical and legal).

**Response:** Since there is a separate mention for the NONAGINTA study approval and written informed consent, we referred to Finnish legislation to clarify that the framework under which the study began was ethical and consistent with legislation at the time.

P8 Table 1 - Why the sudden drop in 1990 to N = 53 (see earlier point - inclusion criteria at this phase not clear, needs STROBE detail). Why APOE-e4 different proportions for men/women?

**Response**: 1975 and 1981 questionnaires were mailed to all twins born before 1958 and living in Finland, identified from the Finnish population register. The 1990 questionnaire was targeted to twins born in 1930–1958 who had responded to either earlier questionnaire. Those born before 1930 were not included in 1990, making the number of participants with data in 1990 lower than other years. In the NONAGINTA (90-year-old twins) data collection, twins born before 1930 were also invited. Regarding APOE-e4, men and women carry the allele at similar frequencies in general population. In this sample, it seems there is a higher proportion of carriers within women. We have no clear explanation for why this is.

P9L179 Healthy survivor effects - very few smokers remained at older ages.

**Response**: Some text about this was added to the discussion section (page 19): “The healthy survivor effect can also influence our results, as individuals who survive to older ages, especially above 90-years, usually represent a subset of a population with better health profiles.”

P12L257 The drop-out analysis is informative but descriptive - it is does not evaluate the impact of bias on substantive results, whereas IPW might to some extent.

**Response:** This is a good notion, and IPW was included for a more informative analysis on the drop-out bias and how it impacts the results.

P12L267 Nothing mentioned on childhood IQ which could drive CVD risk, cognitive aging, dementia risk across the life course. Studies should be cited on this missing antecedent variable.

**Response**: Sentences added to first paragraph in discussion section (page 16-17): “It should be noted that higher childhood intelligence has been associated with both higher old age cognition [26] and lower CV disease risk [27]. We did not have a direct measure for childhood intelligence, which is a limitation in this study. Nevertheless, higher educational attainment is listed as one of the most influential protective factor against dementia in the latest Lancet commission report on dementia risk factors [7].”

P13L277 Be clearer here - are you saying hypertension and higher BMI were associated with better cognitive function (cross-sectionally), less cognitive decline. Longitudinally? Were these impacted potentially by reverse causal, healthy survivor, dropout effects? Are there any administrative data / whole population studies which can clarify or compare?

**Response:** This sentence was clarified and reflection on the impact of healthy survivor bias and reverse causations was added (page 17).

P13L282 Suggest "participants" not "people" here.

**Response**: Corrected

P13L283 Rewrite for clarity: "...is one of the most prevalent dementia risk factors, with nearly 60% increased risk...". I do not understand this sentence. Do you mean being hypertensive at midlife is the most commonly ocurring risk factor, and that aditionally it increases relative risk by 60%, or 60% of dementia is attributable to hypertension at midlife, or something else? Rewrite so that it cannot be misunderstood.

**Response:** This sentence was modified to the following version for clarity (page 18): “Midlife hypertension is one of the most common dementia risk factors, associated with nearly 60% increased relative risk of developing all cause dementia [24].”

P14L294 This section feels a little rushed e.g. "should be considered when interpreting results". Why? Be specific. Lean into your main limitations - low statistical power, healthy survivor effects, multiple testing, lots of predictors, lots of heterogeneity. Position these with clear next steps for future researchers to design precise research questions and better study designs.

**Response**: The following paragraph was added to more deeply reflect on these limitations and the context of the current findings (pages 18-19): “This study's strengths include a 48-year follow-up and a population-based sample with a nearly equal gender balance. There are several limitations in this study which warrant cautious interpretation of our findings. It is important to consider the small sample size and limited statistical power of the regression analyses, which increases the risk of failing to detect true effects. Additionally, including a high number of covariates relative to the sample size can lead to unstable estimates and inflated standard errors, which may limit the reliability and generalizability of the findings. The healthy survivor effect can also influence our results, as individuals who survive to older ages, especially above 90-years, usually represent a subset of a population with better health profiles. This may also generate spurious associations, including reverse causations. No formal correction for multiple testing was applied due to the exploratory nature of the study and the limited sample size, increasing the potential for false positive findings. However, findings were interpreted with caution, considering consistency across cognitive measures and effect sizes rather than relying solely on p-values. Heterogeneity in the risk profiles could influence the findings, as 90-year-olds tend to have a high prevalence of risk factors (e.g., high BP, low PA), diluting differences between individuals [33]. Lastly, telephone based cognitive assessment for the oldest old likely includes more measurement error related to sensory limitations, distraction and comprehension issues compared to younger old adults. A floor effect was observed in delayed recall, but this was accounted for in the statistical analyses. These limitations highlight the need for future research with larger, more representative samples and optimized study designs that enable more precise estimation of effects and reduce the risk of spurious findings.”

Suggestions for future study designs were also mentioned (page 20): “Future research should focus on investigating the contributions of early-life cognitive ability and educational attainment using longitudinal life course data. Pooling data across multiple population-based studies would improve statistical power and allow for better understanding of population heterogeneity in these associations.”

P14L300 This one sentence on 'selected group' understates the problem - it could be a whole paragraph in my view. Also - IPW might fundamentally offer different and more interesting results, even if to show how different the results are when explicitly modelling dropout characteristics using weights.

**Response:** More reflection on this was included (page 18-19), and IPW was conducted.

P14L305 Careful here - you are making policy statements (access to education) but childhood IQ was not measured. In older cohorts, number of years of education would be correlated with childhood IQ but societal factors might drive number of years, particularly for women. You cannot disentangle childhood intelligence from educational attainment easily (and not at all in this dataset), so you should not be making statements about educational policy and resource allocation. Reword as possible mechanisms for future study in longitudinal (ideally life course longitudinal) studies. Also, given low power, you should make the suggestions that studies from several populations should be pooled together, to improve power and study heterogeneity of effects (e.g. individual participant meta-analysis).

**Response:** The conclusion (page 20) was modified according to this comment. The policy statements about education were removed, and early-life cognitive ability was mentioned in association with the findings about higher education and better cognitive function. Suggestions for future research was included in line with these suggestions (see response for earlier comment P14L294).

Figure 1. This needs redesigning slightly - the arrows were confusing me. NONAGINTA was created in 2020/23 but the arrows moving to the right refer to it from 1975 onwards, higher up. Please design so that it is clearer at first glance, that NONAGINTA is nested within a wider cohort. See STROBE guidelines and flow diagram examples. Again, I wasn't clear why the 1990 cohort was so much smaller - is this by design? What is the reason?

**Response**: The flow chart was redesigned.

REVIEWER #1

**Dear Authors,**

Thank you for the opportunity to review your manuscript entitled “Midlife and old-age cardiovascular risk factors, educational attainment, and cognition at 90-years – population-based study with 48-years of follow-up.”

I kindly commend you on an ambitious and important study that uses a rich, population-based twin cohort to investigate the long-term impact of education and cardiovascular health on cognition in the oldest-old. The extensive follow-up and the integration of both midlife and late-life risk factors add considerable value to the field of cognitive aging. Please find below my feedback.

I believe these revisions will substantially strengthen the manuscript. Thank you again for your thoughtful and important contribution to the literature on aging and cognitive health.

With best regards,

The Reviewer

-----

Major Points

1. Sample Size and Statistical Power

• The final sample size (N = 96) is modest, especially for subgroup comparisons (e.g., BP categories), and likely underpowered for some outcomes such as delayed recall.

• Please comment on the statistical power for the primary outcomes. Consider reporting effect sizes (e.g., Cohen’s d or beta coefficients with 95% CI) consistently throughout to contextualize findings.

**Response:** More text was added to the discussion, reflecting on the small sample size and the limited statistical power in some analyses (pages 18-19). Cohen’s d and standardized beta coefficients were added to the text (see results section, pages 12-15, and discussion, pages 16-17) for the statistically significant results to better contextualize findings.

2. Reverse Causation and Selection Bias

• The finding that midlife hypertension is associated with better cognitive outcomes is contrary to existing literature and may reflect reverse causality or selection bias.

• Discuss this thoroughly and consider stratified analyses (e.g., by antihypertensive medication use).

• Consider controlling for or reporting on duration of medication use.

• Acknowledge potential survivor bias more explicitly in both the results and discussion.

**Response:** More reflection was added to the discussion on the findings of midlife hypertension and better old age cognition, and selection bias and other factors are mentioned For example, see page 18: “A sample consisting of individuals who have lived past 90 years old is also likely to introduce survivor bias in the study, resulting in unexpected associations of the midlife and old age risk factors,“ and page 19: “The healthy survivor effect can also influence our results, as individuals who survive to older ages, especially above 90-years, usually represent a subset of a population with better health profiles.” We did not have enough participants who reported having used antihypertensive medication in midlife (4 in 1975, and 6 in 1981) so further analyses were not conducted. Descriptive information on the medication use was included. We modified the discussion section to emphasize the role of survivor bias more (page 17-18).

3. Interpretation of CAIDE Score

• The CAIDE score did not predict cognitive outcomes, despite being a validated dementia risk tool. This discrepancy is insufficiently addressed.

• Discuss potential reasons why CAIDE performed poorly.

• Clarify how CAIDE scores were calculated from self-report.

• Consider additional analyses excluding the education component or including APOE status.

**Response:** More text was added discussing the potential reasons behind the findings related to CAIDE score (see discussion, page 17-18): “The CAIDE score was originally developed to predict 20-year dementia risk, and it might not be appropriate for predicting cognitive function at age 90, or outcome based on cognitive tests. A sample consisting of individuals who have lived past 90 years old is also likely to introduce survivor bias in the study, resulting in unexpected associations of the midlife and old age risk factors. It is also possible that cognitive risk factors after 90 differ from those affecting younger older adults (60+), as previous studies suggest [23]. “

We also included a more extensive explanation on how the CAIDE score was calculated from self-report (page 6). All CAIDE analyses also included CAIDE variable without education, and models with and without *APOE* status.

4. Educational-Occupational Score

• This composite measure consistently predicted cognition, outperforming CAIDE.

• Clarify what time period the work-related questions refer to. Discuss the extent to which this score may reflect cognitive reserve, and how it relates to lifelong intellectual engagement.

**Response:** The time period and mean age at which the work-related questions were measured was clarified in the methods (see page 7). More text was added to the discussion (see page 16), reflecting on the educational-occupational score’s link to cognitive reserve and intellectual engagement throughout the lifespan: “The educational-occupational score can be considered as a proxy of cognitive reserve, reflecting both lifelong exposure to intellectually demanding environments and possibly also young age cognitive ability [12,24,25]. The educational-occupational score might be more directly linked to intellectual engagement and resilience against age-related cognitive decline, compared to CAIDE which emphasizes vascular health. It should be noted that higher childhood intelligence has been associated with both higher old age cognition [26] and lower CV disease risk [27]. We did not have a direct measure for childhood intelligence, which is a limitation in this study.”

5. Delayed Recall Floor Effects

• The average delayed recall score (2.2 out of 10) suggests a potential floor effect.

• Consider alternative modeling strategies (e.g., zero-inflated or transformed models).

• Discuss limitations of the telephone-based assessment in very old adults.

**Response:** We agree that delayed recall measure was skewed towards zero values. This is why we used negative binomial regression for all delayed recall analyses, a method that is suitable for this type of variable. A mention of the floor effect and percentage of people with score of zero was added to the results and discussion sections (page 11 and page 18). Limitations introduced by the telephone-based cognitive assessment for the oldest old was added to the end of the discussion (page 18): “Lastly, telephone based cognitive assessment for the oldest old likely includes more measurement error related to sensory limitations, distraction and comprehension issues compared to younger old adults.” We also note that based on other comments, we have now included analyses with composite score in the revised manuscript; this variable includes also delayed recall measure and does not have a skewed distribution (see response to editor comment #7).

Minor Comments

• Abstract: Consider rephrasing “no consistent associations” with more precise language.

**Response:** The sentence was rephrased to “were not significantly associated with cognition at 90+ years.”

• Figure captions: Add sample sizes and clarify whether multiple comparisons were corrected.

**Response:** The sample sizes vary between each year and each risk factor, so adding them to the figure or figure caption made interpretation complicated. We added a mention to the figure captions, specifying the sample size range and that the sample sizes can be seen in the regression tables in the supplement.

• Define what constitutes “high” vs. “normal” BP based on Finnish guidelines in the 1970s–1980s.

**Response:** A sentence was added to the methods section (page 5), “Finnish guidelines for hypertension thresholds in 1970-1980s were set at 160/95 to 175/100 mmHg [15,16].”

• Specify how missing data were handled (e.g., imputation, complete-case analysis).

**Response:** For education variable, missing data was filled in from previous questionnaires when available, and if not, the median was imputed. For mean BMI and mean MET from 1975 and 1981, if only one year was available single measure was used. For all other variables (BP, cholesterol) only complete cases were included. There were no missing cases for age, sex, and follow-up time data, or for cognitive measures. This information is also mentioned in the supplementary text. A sentence was added to the Methods section to clarify we used complete cases (page 8): “Only complete cases were included in the analyses.”

• Clarify APOE genotyping procedures (e.g., DNA source, platform).

**Response:** Following text was added to the supplement (referred to in the main text, page 8): “APOE genotypes were derived from the DNA samples (blood, saliva) of the Old Finnish Twin Cohort participants which were collected during several studies. The samples, along with signed consent forms, were returned to the Finnish Institute for Health and Welfare (until 2008 called National Public Health Institute) in Helsinki, Finland, for Apolipoprotein *E* (*APOE*) genotyping. Genome-wide genotyping arrays (Illumina) were used, and genotype data were run through several quality control phases prior to pre-phasing and imputation. The imputation was performed using Haplotype Reference Consortium reference panel consisting of ~32000 European-ancestry samples resulting ~32 million variants. The whole process of imputing APOE genotypes with comparison using different reference panels has been earlier published elsewhere (5).” (<https://pmc.ncbi.nlm.nih.gov/articles/PMC6991323/>).

Questions for the Authors

1. Can you elaborate on how education was treated longitudinally—was the highest value used across waves?

**Response:** Education measured at the time of the risk factor was used, and filled in with most recent questionnaire if missing. This is mentioned in the methods section (page 6).

2. Could the paradoxical effect of midlife hypertension reflect early treatment or better access to care? Have you considered stratifying analyses based on antihypertensive use?

**Response:** This is an excellent point, and we agree that the paradoxical association of high BP in midlife and better cognition at 90 years old could reflect a selected group who were aware of their high BP and could have had better access to services. They would also be more likely to use antihypertensive medication than those who were not aware of having high BP. Following text is included in the discussion (page 18): “The earlier start of antihypertensive medications could explain the unexpected association between high BP in midlife and better cognition in very old age. Considering the small sample size and low number of people in the high BP groups, it is possible these findings reflect reverse causality or selection bias. In 1970s Finland, many were unaware of having high BP [32], and some participants may have been misclassified as having normal BP, while those on medication might represent a group with better access to services.”

We considered analyses with antihypertensive use, but the sample sizes for those who reported having used antihypertensive medication for more than 10 days in the past year in 1975 or 1981 were too small for meaningful analyses (n=4 and n=6). As a covariate a variable with such small groups could result in unstable estimates. Descriptive information on the antihypertensive medication use was included in the manuscript (page 18) and the supplement.

3. Do you view the educational-occupational score as a proxy for cognitive reserve? If so, how does this fit with the null CAIDE findings?

**Response:** We do consider the educational-occupational score to reflect the level of cognitive reserve to at least some extent. Some reflection about this was added to the manuscript (see earlier response to comment #4).

4. Do attrition analyses suggest meaningful differences in baseline risk factors between completers and non-completers?

**Response:** The results from the drop-out analyses were generally as expected, the study participants had higher educational and occupational attainment, and slightly better health profiles reflected in the significant differences in CAIDE scores. To address if these differences impacted the study results, we conducted IPW attrition analyses, as suggested in this revision. The results from the weighted regressions remained consistent with the original findings.

5. How might findings generalize to more diverse, less homogeneous populations?

**Response:** This is an excellent question, and a meaningful avenue for future studies. Although our study sample was a population-based cohort, and reflected the general population in Finland, the participants tend to be more highly educated and have healthier lifestyle. There was not much diversity in terms of ethnicity, which could also limit the generalizability of these findings.

REVIEWER #2

This study examined the association between cognitive function and cardiovascular risk factors. 96 participants were followed up for an average of 46 years. High numbers of statistical test were performed and the main result showed that high education was associated with better cognitive functioning at 90 years of age. Some other associations were also found but these remained a bit inconsistent. Interestingly, CAIDE score was not associated with cognitive function.

The long follow-up time is the greatest strength of this study. However, the number of participants is quite low, N=96. This is a significant concern that brings a lot of uncertainty to the interpretation of the results. Low number of participants and high number of statistical tests raises the concern of multiple testing problem.

Furthermore, I have some other comments.

1. What were the shortest and the longest follow-up time?

**Response:** From baseline, the follow-up time ranged between 45-48 years. This was added to the descriptive statistics section in page 9.

2. In covariates, blood pressure and total cholesterol were used as binary variables where high and normal levels were evaluated based on self-reported questionnaire. This raises some concerns because, for example, very low blood pressure has been reported to be associated with poor cognitive function or increased dementia risk. The self-reported high or normal levels may increase the risk of bias because the cut-off values for the binary variables are not known at all. It would be clearer to state in the actual manuscript that the covariates are not based on actual measurements.

**Response:** A sentence was added to clarify BP was based on a self-report measure (see page 5). Detailed questions on which the BP variable was based on can be seen in supplement. The guidelines at the time for hypertension levels were also included (page 5): “This was based on a self-report measure from the questionnaires (see the Supplement, S2 Text). Finnish guidelines for hypertension thresholds in 1970-1980s were set at 160/95 to 175/100 mmHg [15,16].”

3. Please address the potential role of multiple testing on the observed associations.

**Response:** The following section was added to the manuscript (see page 18, Discussion) mentioning the issue of multiple testing and how it could affect the observed associations: “No formal correction for multiple testing was applied due to the exploratory nature of the study and the limited sample size, increasing the potential for false positive findings. However, findings were interpreted with caution, considering consistency across cognitive measures and effect sizes rather than relying solely on p-values.”

4. There are no details on how many of participants had dementia. How are the risk factors associated with dementia risk?

**Response:** Nine participants reported having Alzheimer’s disease and two participants reported having other dementia causing neurodegenerative disease. These were based on self-reports asking if their doctor had told them they had dementia causing neurodegenerative disease. Cardiovascular risk factors have been associated with dementia risk in previous studies. In our study, we wanted to focus on cognitive outcome variables, which capture cognitive domains that have been associated with early changes in the Alzheimer’s disease continuum; this way we have potential to capture subtle changes in cognitive function.
